# Palliative care in urgent need of recognition and development in general practice: the example of Germany

**DOI:** 10.1186/1471-2296-11-66

**Published:** 2010-09-15

**Authors:** Nils Schneider, Geoffrey K Mitchell, Scott A Murray

**Affiliations:** 1 End-of-life Care Research Group, Institute of Epidemiology, Social Medicine and Health System Research, Hannover Medical School, Hannover, Germany; 2 Discipline of General Practice, University of Queensland School of Medicine, Queensland, Australia; 3 Primary Palliative Care Research Group, Centre for Populations Health Sciences, The University of Edinburgh, Edinburgh, UK

## Abstract

**Background:**

Specialist palliative care is being increasingly recognised and developed to improve end-of-life care in many developed countries. However, only a small proportion of the total number of patients with incurable, progressive diseases actually has direct contact with specialist palliative care practitioners. Using the German situation as an example, the main purpose of this paper is to argue that the emphasis on specialist palliative care services without a similar encouragement of primary palliative care will deliver a constrained service.

**Discussion:**

For the vast majority of people with incurable, progressive diseases, good palliative care delivered by General Practitioners and community nurses, with access to specialist support when needed, is the optimal response. In Germany, specialist palliative care in the community was established in the 2007 health care reforms. However actual and potential delivery of palliative care by general practitioners and community based nurses has been sorely neglected. The time-consuming care of palliative patients and their families is currently far from accurately reflected in German, indeed most European primary care payment systems. However, it is not just a question of adequate financial compensation but also of the recognition of the fundamental value of this intense form of holistic family medicine.

**Summary:**

It is imperative palliative care carried out by community nurses and general practitioners is better recognised by health professionals, health insurers, government and the scientific community as a central part of the delivery of health care for people in the last phase of life. Health systems should be arranged so that this critical role of general practice and primary care is intentionally fostered. Palliative care carried out by generalists needs an identity at an academic and practical level, developing in concert with specialist palliative care.

## Background

Delivering appropriate care for people with incurable progressive diseases in the last phase of life is an important, but largely neglected, role of the health system in many countries [1,2]. In recent years deficits in this field have increasingly come to the attention of the public, politicians and professionals, as have insistent demands for the development of palliative care.

Measured in terms of the number of palliative care units, hospices and outpatient palliative care services (palliative care teams), specialist palliative care has grown considerably. However, according to expert estimates, the need remains far from met [3]. This is particularly true for care in the community. Although most people want to die at home, only about 30% are successful in doing so, with the majority dying in hospitals or in nursing homes [1,4].

In Germany, for example, in order to enable more people to spend the last phase of their lives at home, avoid unnecessary hospital admissions and improve patients' quality of life, legislators established specialist palliative care in the community (*spezialisierte ambulante Palliativversorgung, SAPV*) as part of the 2007 health care reforms (§ 37b of German Social Security Code V), with varying degrees of specialist support, ranging from one-off consultations to full palliative care provided by a specialist team. By the end of 2009, approximately 50 SAPV contracts between the Statutory Health Insurance and service providers had been agreed nationwide [5]. The movement to implementation is in practice sluggish, however, and is complicated by unanswered questions regarding requirements, content and structural design, and by problems in quality assurance and resource distribution. These implementation problems should not detract from the promising potential SAPV has in bringing about sustainable improvement through innovative approaches to caring for the seriously and terminally ill. The introduction of SAPV is in line with the international trend towards specialist palliative care services for patients with particularly high care needs [1,2].

## Discussion

The fact should not be overlooked that even if specialist palliative care became fully available in the future, only a relatively small proportion of the total number of patients with incurable, progressive diseases will benefit from this care. It is estimated that 10% of the incurable, seriously ill and dying need some form of specialist palliative care [6]. The vast majority of those affected do not require specialised care, but can be adequately treated within primary care, some with varying degrees of specialist support. Internationally, the phrase 'primary palliative care' is generally used as a generic term for the activities of GPs and home care nursing services in this field [7].

### Potential of GPs

General practitioners have the potential and ability to provide end-of-life care for most patients, given adequate training, resources and, when needed, specialist advice [8,9]. The introduction of SAPV meets requirements for specialist advice, and the majority of family doctors welcome this opportunity: in a survey of GPs in Lower Saxony, 56% indicated the likelihood that they would, in relevant cases, consult SAPV teams as "definite" or "probable", and the same number could imagine themselves sharing the delivery of patient care with an SAPV team. Only a minority of 15% can imagine entirely handing over the care of their palliative patients [10]. These results underline the self-perception of general practitioners that they support their patients continuously through various stages of life, assisting in a variety of care situations right up until death.

The level of family doctors' commitment to seriously ill and dying patients in their practices is also demonstrated by the fact that around half of GPs indicate that they are, regardless of the official rules of the physicians' emergency service, always available [i.e. around the clock] to their palliative patients - sometimes personally, sometimes through informal arrangements with colleagues. This willingness seems to become more pronounced as the patient's death comes closer [11].

For patients and relatives, the family doctor is the primary contact, including - and especially - in the case of an incurable, progressive disease [12]. The doctor-patient relationship, ideally developed over years, together with the focus on whole patient care with a biopsychosocial approach which is characteristic of the general practice, means that family doctors have the inestimable advantage of knowing palliative patients even before the onset of the incurable disease, and so proactively influence the care provided. This is an advantage GPs have over specialist palliative care physicians, who have a relatively late first contact with patients and relatives.

### Different illness trajectories

Forty-two per cent of people die aged between 60 and 80, and 44% die at over 80 years of age. With advancing age, cardiovascular disease as a principal cause of death is more relevant than malignant neoplasm [13,14]. Elderly people with dementia, frailty or multiple chronic conditions formulate the largest group of patients in GPs' practices, responsible for around 35% of deaths, and an even greater percentage of the need for palliative care as their illness trajectories of decline are relatively long [15].

As in other European countries, hospices, palliative care units and palliative care teams in Germany focus on care for cancer patients. In 2008, 90% of the patients cared for by hospices and palliative care units, and 74% cared for by palliative care teams in the community suffered from cancer. Only approximately 20% had cardiovascular diseases as the leading diagnosis [16]. A number of factors have contributed to this, such as institutional affiliations and financial incentives. One significant reason may be found in the fact that remaining life expectancy and the course of the disease are often relatively easier to estimate in cancer patients than in patients with other incurable, progressive diseases, and so organisation of, and therefore access to specialized care is, comparatively, easier to arrange.

So far three distinct illness trajectories have been described for people with incurable, progressive chronic illnesses: a trajectory with steady progression and usually a clear terminal phase (mostly cancer); a trajectory (for example, respiratory and heart failure) with gradual decline, punctuated by episodes of acute deterioration and some recovery, with more sudden, seemingly unexpected death; and a trajectory of prolonged frailty, poor function, and gradual decline (typical of frail elderly people or people with dementia) [17]. Thus in Germany as in the UK, the palliative care needs of non-cancer patients are often not adequately recognized [e.g. [7,18,19]]. WHO advocacy for better palliative care for older people [20] is a wake up call for groundbreaking action that general practitioners are well placed to take forward internationally.

### Increased demand for primary palliative care

In order to meet these challenges, the development of existing specialist palliative care is an important step. The debate will however fall short of the issue if, as is frequently the case, it focuses almost exclusively on specialist care. For the vast majority of people in the final phase of life with incurable, progressive diseases, good primary care delivered by GPs and community nurses, i.e. primary palliative care, is the optimal response [7]. In politics, public and professional circles, however, this aspect has been sorely neglected.

Beneficial as the introduction of specialist palliative care in the community (SAPV) in Germany was, the failure to encourage primary palliative care at the same time remains baffling. The time-consuming care of palliative patients and their families is currently far from accurately reflected in the primary care compensation system. The same holds for the numerous home visits that are often required, or for the willingness of many primary care doctors to be, for the sake of their patients, available outside of, and in addition to, their official practice times and emergency duties. This is not just a question of adequate financial compensation, but also of the recognition of requirements in health care delivery, and patients' needs, and of the fundamental value of this intense form of holistic family medicine.

### Transition between primary and specialist palliative care

An ideal health system for managing palliative care would see a seamless transition between primary and specialist palliative care, and provision of support in the form of access to evidence based guidelines, supported by specialist involvement ranging from one-off consultations to full referral. In effect, the care of the patient is shared between the two, with the primary care team usually determining when and how much support is required.

**Figure 1:**
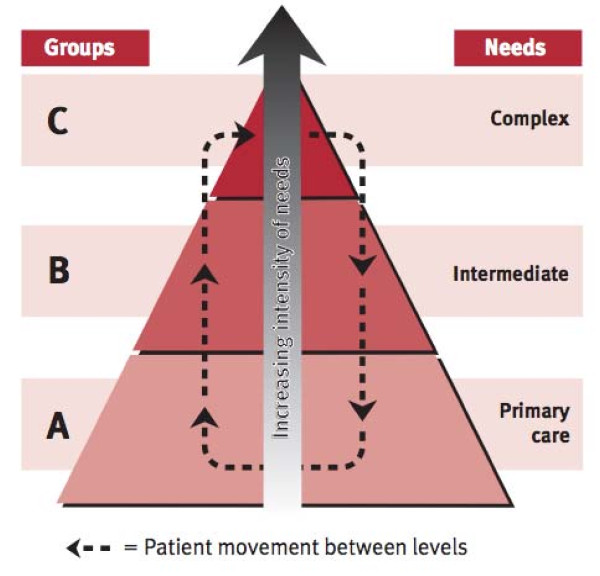
**Seamless transition between specialist and generalist palliative care services according to intensity of need**. [23]

**Figure 2:**
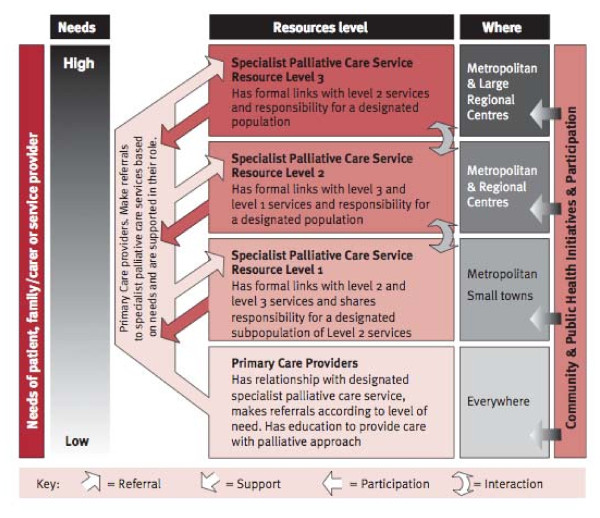
**Organisation of health system to facilitate needs-based palliative care**. [23]

In Australia, for example, much work has been done to facilitate needs-based palliative care delivery to ensure that patients receive the right level of care at the right time [21,22]. A schematic representation of this model developed by Australian policy makers is shown in Figures 1 and 2[23].

In Britain, which has long-established and well developed specialist palliative care services, a general practice-based system of routinely identifying patients who could die, and developing care plans for these people on an individual basis is encouraged by formal financial support by the National Health Service [24,25].

In Germany, specialist palliative care in the community (SAPV) as established with the 2007 health care reforms provides the appropriate legal and system requirements for smooth transition between primary and specialist palliative care [10]. It is much to be hoped that further refinement of the implementation into practice will facilitate and support appropriate primary palliative care.

## Summary

It is imperative that in Germany as well as in other developed nations *primary *palliative care is better recognised by health professionals, health insurers, government and the scientific community as a central part of the delivery of health care for people in the last phase of life. This needs to include academic and conceptual development of the field with appropriate research activities, in order to better determine requirements of education of professionals, and provision of patient and carer-centred care in the community. *Primary *palliative care needs a parallel research and development with specialist palliative care to permit universal access to end of life care.

## Competing interests

The authors are all general practitioners and belong to the International Primary Palliative Care Research Group http://www.uq.edu.au/primarypallcare.

## Authors' contributions

NS conceived the idea for the paper and drafted the manuscript. GM and SM made substantial contributions to conception and equally helped to draft the manuscript. All authors read and approved the final manuscript.

## Pre-publication history

The pre-publication history for this paper can be accessed here:

http://www.biomedcentral.com/1471-2296/11/66/prepub
